# α_S1_-Casein-Loaded Proteo-liposomes as Potential Inhibitors in Amyloid Fibrillogenesis: *In Vivo* Effects on a **C. elegans** Model of Alzheimer’s Disease

**DOI:** 10.1021/acschemneuro.3c00239

**Published:** 2023-10-17

**Authors:** Angela Paterna, Pamela Santonicola, Giulia Di Prima, Estella Rao, Samuele Raccosta, Giuseppina Zampi, Claudio Russo, Oscar Moran, Mauro Manno, Elia Di Schiavi, Fabio Librizzi, Rita Carrotta

**Affiliations:** † Institute of Biophysics, National Research Council, Division of Palermo, Via Ugo La Malfa 153, 90146 Palermo, Italy; ‡ Institute of Biosciences and Bioresources, Division of Napoli, Via Pietro Castellino 111, 80131 Napoli, Italy; § Department of Biological, Chemical and Pharmaceutical Sciences and Technologies, University of Palermo, 90123 Palermo, Italy; ∥ Institute of Biophysics, National Research Council, Division of Genova, Via De Marini 6, 16149 Genova, Italy; @ Department of Medicine and Health Sciences, University of Molise, 86100 Campobasso, Italy; ⊥ Consorzio Interuniversitario in Ingegneria e Medicina (COIIM), Via F. De Sanctis, 86100 Campobasso, Italy

**Keywords:** intrinsic disordered protein, α_S1_-Casein, amyloid inhibition, *C. elegans*, Alzheimer disease, drug delivery, proteo-liposomes

## Abstract

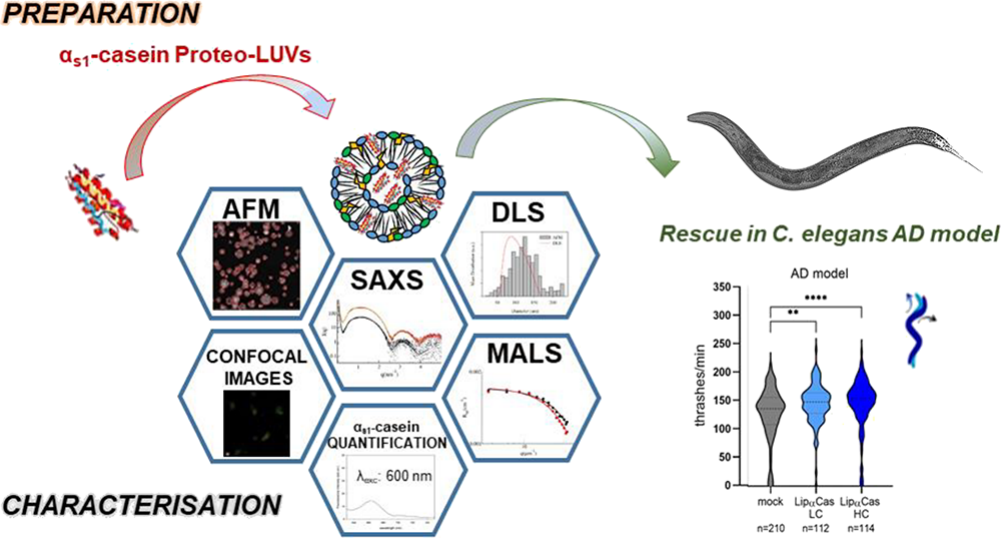

According to the amyloid hypothesis, in the early phases of Alzheimer’s disease (AD), small soluble prefibrillar aggregates of the amyloid β-peptide (Aβ) interact with neuronal membranes, causing neural impairment. Such highly reactive and toxic species form spontaneously and transiently in the amyloid building pathway. A therapeutic strategy consists of the recruitment of these intermediates, thus preventing aberrant interaction with membrane components (lipids and receptors), which in turn may trigger a cascade of cellular disequilibria. Milk α_s1_-Casein is an intrinsically disordered protein that is able to inhibit Aβ amyloid aggregation *in vitro*, by sequestering transient species. In order to test α_s1_-Casein as an inhibitor for the treatment of AD, it needs to be delivered in the place of action. Here, we demonstrate the use of large unilamellar vesicles (LUVs) as suitable nanocarriers for α_s1_-Casein. Proteo-LUVs were prepared and characterized by different biophysical techniques, such as multiangle light scattering, atomic force imaging, and small-angle X-ray scattering; α_s1_-Casein loading was quantified by a fluorescence assay. We demonstrated on a **C. elegans** AD model the effectiveness of the proposed delivery strategy *in vivo*. Proteo-LUVs allow efficient administration of the protein, exerting a positive functional readout at very low doses while avoiding the intrinsic toxicity of α_s1_-Casein. Proteo-LUVs of α_s1_-Casein represent an effective proof of concept for the exploitation of partially disordered proteins as a therapeutic strategy in mild AD conditions.

## Introduction

Alzheimer’s disease (AD) is the most common form of neurodegenerative disorder affecting several tens of millions of elderly people worldwide today, according to the World Health Organization. It therefore constitutes an important medical issue and economic burden for modern societies, in which the average population age continues to grow.^1^ The GBD 2019 Dementia Forecasting Collaborators recently estimated that the number of people with dementia will possibly increase globally from 57 in 2019 to 153 million cases in 2050.^2^ Despite the enormous research efforts, AD remains mostly uncurable, claiming further intensive research on the molecular mechanisms of the disease and possible defusing. In addition to drugs that partially treat symptoms, a human monoclonal antibody (aducanumab), which may change disease progression, was approved in June 2021 by the US Food and Drug Administration (FDA) to treat patients with AD.^3^ However, two trials in phase 3 were halted because no remarkable success in improving cognitive functions was observed.^4^ Very recently, in January 2023, Lecanemab, a humanized IgG1 monoclonal antibody gained accelerated approval by FDA to treat mild AD patients.^5^ However, confirmatory and longer trials are ongoing due to some safety concerns.

The origin of neurodegeneration in AD is still debated although, as for other neurodegenerative disorders, the involvement of amyloid aggregation in the disease is established.^6,7^ Amyloid aggregates arise from the misfolding of proteins and/or the formation of metastable oligomers and their successive arrangement into highly ordered intermolecular β-sheet structures, called amyloid fibers, which can reach hundreds of nanometers or even micrometers in length. Aberrant amyloid deposits are always found in post-mortem inspection of the brain of AD patients. These deposits are mainly constituted by neurofibrillary tangles of tau protein and amyloid plaques composed of 37–42 residue peptide, the amyloid β-peptide (Aβ), derived from the proteolysis of the Amyloid Precursor Protein (APP).^8,9^ According to the amyloid hypothesis, small oligomers or protofibrils of Aβ peptides can be highly neurotoxic and ultimately produce neurodegeneration and loss of cognitive functions.^10,11^ In this framework, the retrieving of methods able to interfere with the amyloid formation and reduce its effects in living systems can help in the design of new strategies against AD. Indeed, the recently approved antibody Lecanemab recognizes Aβ soluble protofibrils, delaying the progress of cognitive impairment in mild AD patients.^5^

In recent years, several systems have been shown to inhibit amyloid formation to different degrees.^6,12−17^ Among these, bovine α_s1_-Casein has proven to be very effective against Aβ_40_ aggregation, even at very low molar ratio.^18^ The capability of α_s1_-Casein to prevent Aβ amyloid formation is related to its intrinsically disordered nature with the inherent solvent exposure of hydrophobic patches capable of sequestering aggregation-prone Aβ species. More specifically, α_s1_-Casein is highly effective even at extremely low concentrations and ratios relative to Aβ, likely due to the double nucleation (homogeneous and heterogeneous) behavior of the Aβ amyloid formation. In fact, as for other amyloidogenic proteins, the aggregation of Aβ is characterized by the existence of secondary nucleation mechanisms, such as fragmentation or surface nucleation, for which the presence of small quantities of already formed amyloid aggregates can exponentially catalyze the formation of new aggregates.^19^ In such a situation, the ability of α_s1_-Casein to sequester early aggregates before they become large enough to trigger secondary nucleation may result in a strong inhibition of the overall aggregation process even at a very low inhibitor concentration. Indeed, it has been shown that α_s1_-Casein has a partial effect on the aggregation process of proteins not characterized by secondary nucleation mechanisms.^20^ On the contrary, the inhibiting effect of α_s1_-Casein is remarkable^21^ on systems in which amyloid formation is strongly influenced by secondary nucleation processes, such as insulin.^22−25^ In this respect, the α_s1_-Casein mechanism of amyloid fibrillogenesis inhibition is similar to the one recognized for the chaperonin Hsp60.^15,16,26^

The same properties that enable α_s1_-Casein to prevent amyloid aggregation, i.e., its disordered structure and amphipathic nature, hamper its use in living systems. Indeed, α_s1_-Casein forms micelles above its critical micellar concentration. Furthermore, even at a lower concentration, it easily adheres to surfaces, making problematic many common laboratory treatments, such as labeling, filtering, reconcentrating, etc. In this context, its function to hinder amyloid formation in vivo can be promoted by suitable carriers capable of preventing the formation of micelles and the nonspecific adhesion to surfaces and cellular membranes. Among the variety of existing drug delivery strategies, the encapsulation of the active agent into biocompatible nanosystems is extremely promising. For instance, recent work shows that β-casein coated gold-nanoparticles can cross the brain-blood barrier in zebrafish larvae and to sequester in vivo intracerebral Aβ_42_ injected in the larvae brain, successfully preventing the toxic effects observed in their absence.^27^ The use of animal models can be fundamental to identifying treatments impacting Aβ formation in vivo. The nematode **C. elegans** is a powerful model system, widely used to study the genetic basis of AD and Aβ toxicity.^28^ Even if in **C. elegans** the cleavage of APL-1, the APP ortholog, does not produce Aβ peptide,^29^ many transgenic strains have been generated to express the human Aβ peptide in all cells, or specifically in neurons and muscle cells.^30^ Interestingly, Aβ peptide expression in **C. elegans** causes aggregates accumulation and age-dependent defects in locomotion.^31,32^ Moreover, recent studies demonstrated the power of **C. elegans** to successfully screen AD drug candidates^33^ and to validate the efficacy of nanoconjugate drug delivery systems.^34^ As nanocarriers, unilamellar liposomes are lipid-based vesicular systems, which emerged over the past 30 years due to their biocompatibility, versatility, and capability of encapsulating both hydrophobic and hydrophilic molecules to be delivered to cells^35,36^ or to animal models.^37^

The aim of this work was to prove the concept that vehiculating α_s1_-Casein with liposomes improves its therapeutic potential in terms of mitigating the detrimental effects caused by amyloid aggregation. Here, we explore the possibility to encapsulate α_s1_-Casein in suitable large unilamellar lipid vesicles (LUV) made by extrusion and using them in vivo as carriers on a **C. elegans** AD model. α_s1_-Casein-loaded proteo-LUVs were produced by coextrusion of a POPC:POPS:cholesterol lipid mixture with the protein. In this way, the partially disordered structure of α_s1_-Casein molecules could be incorporated both in the internal cavity of liposomes and in their bilayer structure. The proteo-LUV (LipCas) and the *solo* LUV (Lip0) were characterized by different biophysical techniques, namely, dynamic light scattering (DLS), atomic force microscopy (AFM), and small-angle X-ray scattering (SAXS). The characterization highlights that α_s1_-Casein significantly participates in the formation of the proteo-LUV dimension by being likely exposed on the surface, as evidenced by the increased mean size and the adhesion properties of the nanoparticles on mica and by the signature of the bilayer. Then, to demonstrate in vivo the effects of α_s1_-Casein and the importance of its delivery through liposomes, we tested them on a *C. elegans* AD model expressing human Aβ in muscles. We initially observed a reduction of Aβ aggregates and the rescue of the locomotion defects of the AD model animals when α_s1_-Casein is administered. Then, we demonstrated that proteo-LUV is able to efficiently deliver subnanomolar α_s1_-Casein doses, eliminating any side effects due to the administration of the protein at higher doses.

## Results and Discussion

α_s1_-Casein is a 24 kDa naturally occurring protein, included in the family of intrinsically disordered proteins (IDP), chemically constituted by two highly hydrophobic segments separated by a hydrophilic moiety based on seven phosphate groups.^38,39^ Its amphiphilic nature makes it capable of self-assembling above 0.21 mg/mL in the conditions here used. The knowledge of the critical micelle concentration (CMC) value under the conditions of the study was crucial to decide a fixed protein concentration, below the CMC, to prepare the proteo-LUV and to employ the highest amount of protein before aggregates formation. Due to α_s1_-Casein characteristics, large unilamellar vesicles resulted in the most suitable carrier to encapsulate the protein as they are big enough to incorporate such large molecules (e.g., proteins, mRNA, etc.) and able to interact with both hydrophilic and hydrophobic regions of the protein. Proteo-LUV were thus prepared from multilamellar samples obtained by hydration of the lipid film with a 0.2 mg/mL α_s1_-Casein solution. The lipid composition was chosen to mimic the charge of the cell membrane (PC:PS in 9:1 ratio) while lipid saturation together with cholesterol concentration contribute to obtaining a liquid crystal phase matrix at room temperature and over.^40^ Additionally, the chosen lipid composition strictly resembles the neuronal membrane composition, thus surely leading to high biocompatibility.

### Biophysical Evaluation of α_s1_-Casein Proteo-LUV

LUV and proteo-LUVs were prepared without (Lip0) and with α_s1_-Casein (LipCas), respectively, and then characterized by different biophysical techniques to study the differences due to the presence of the protein and evaluate the interaction between the protein and the lipid matrix.

#### Size Distribution

**Figure 1:**
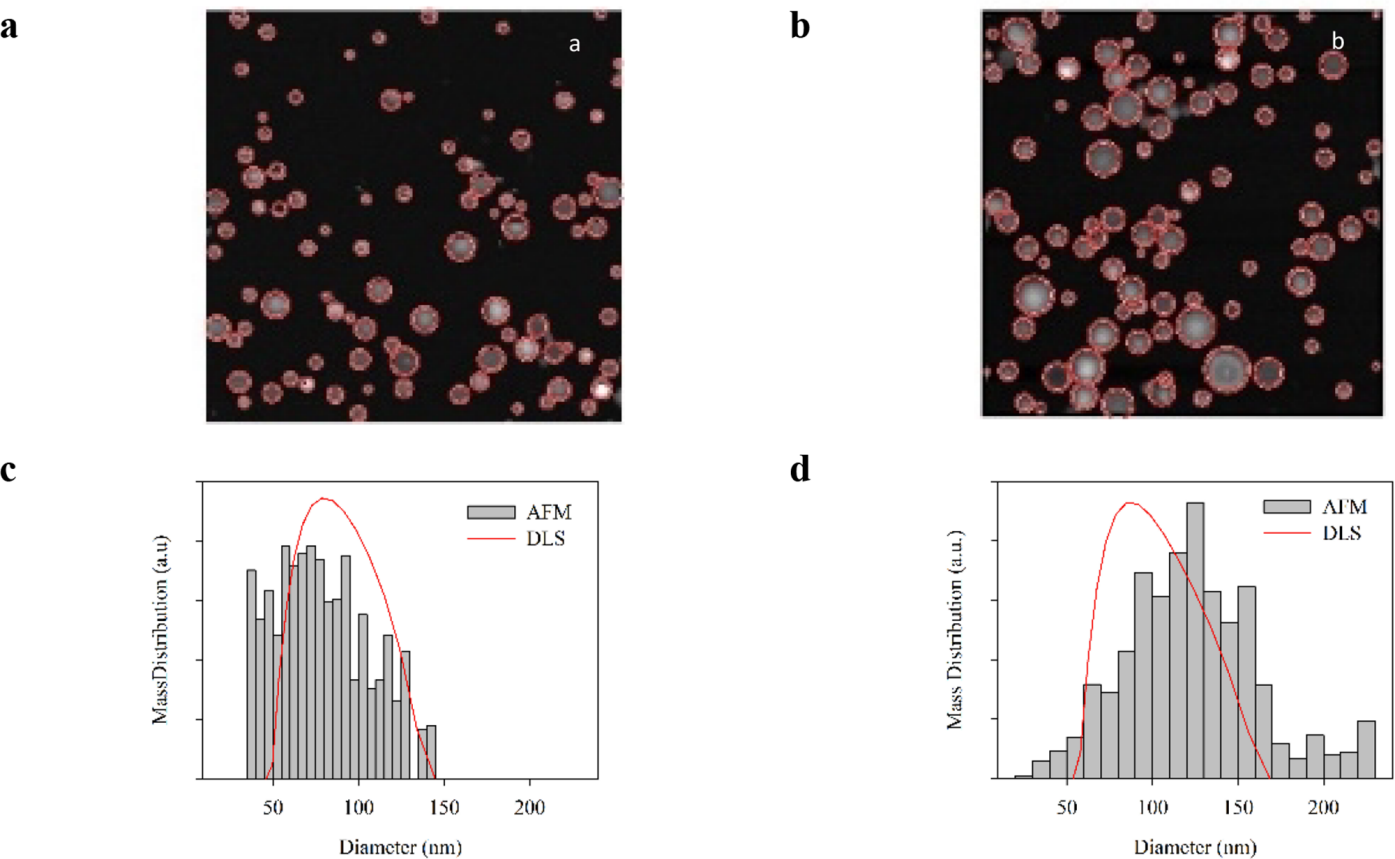
AFM scan and particle analysis for (a) Lip0 (first scan) and (b) LipCas (second scan). Mass weighted size distributions for Lip0 (c) and LipCas (d), obtained by AFM (histogram) and dynamic light scattering (red line).

LipCas were prepared by the extrusion method by using a 50 nm filter to obtain a size suitable for drug delivery. The size distribution of the samples after preparation was tested by dynamic light scattering and by atomic force microscopy (AFM). Concerning AFM measurements, it is important to highlight that the liposomes could be optimally adsorbed on the mica surface only when preliminarily diluted at pH 5. Interestingly, in the absence of this predilution step, a difference between Lip0 and LipCas was observed, since α_s1_-Casein proteo-LUV showed a stronger interaction with mica (data not shown). At low pH, a residual tendency to be dragged by the AFM tip was still observed for both liposomes, despite the use of the Quantitative Imaging mode, which minimizes drag forces; again, this tendency was sizably stronger for Lip0. Due to the presence of POPS in the matrix, the LUV have a negative net charge at neutral pH and the repulsive electrostatic forces make it difficult for their adhesion on freshly cleaved mica surface. Dilution of LUV in an acidic buffer reduces their net charge thanks to the partial neutralization of serine groups facilitating the adhesion on mica by nonspecific interactions. For LipCas, the presence of α_s1_-Casein favors the interaction of the proteo-LUV with the mica surface, strongly suggesting that the protein due to its amphiphilic nature is interacting with the bilayer, modifying the liposomes surface.^41^ In all conditions, Lip0 resulted in much less resistance and adhesiveness to the mica surface than LipCas. In fact, in the case of Lip0, we observed a strong tendency to detach from mica after scanning, which limited the possibility of imaging the sample only to the first scan; in contrast, in the case of LipCas, due to an improved adhesion to mica, consecutive scans were always possible (see Figure 1a,b where the first scan for Lip0 and the second scan for LipCas, respectively). Image analysis performed through a homemade recognition program^42^ provides the size particles distribution for the two samples, reported in Figure 1c,d together with the analogous size distributions obtained in solution from DLS (for the sake of comparison, mass-weighted distributions for both techniques are reported). On average, LipCas nanoparticles are larger, indicating the presence of part of the protein on the shell, in agreement with the different adhesion properties.

#### Surface Hydration

The amphiphilic nature of α_S1_-Casein inspired the design of proteo-LUV and the idea of its incorporation into the lipid bilayer space. In this view, the Laurdan assay gives information about differences in the relative hydration of the bilayer due to the interaction of the protein with the bilayer. The different surrounding environment, as seen by Laurdan molecules, affects its emission spectrum, with a red-shift observable moving toward more polar environments. These variations can be expressed by calculating the Generalized Polarization (GP) by the Laurdan emission spectrum (see methods), by considering the blue (435 nm) and the red (500 nm) edge of the spectrum.^43^ GP values vary between −1 and 1, going from completely polar to apolar environments. For our samples, the obtained GP values were 0.268 ± 0.016 and 0.185 ± 0.011 for Lip0 and LipCas, respectively. These results indicate that casein, in agreement with AFM and DLS data, is also located in the bilayer to some extent, thus slightly increasing the hydration of the bilayer surface.

#### Structural Information

**Figure 2:**
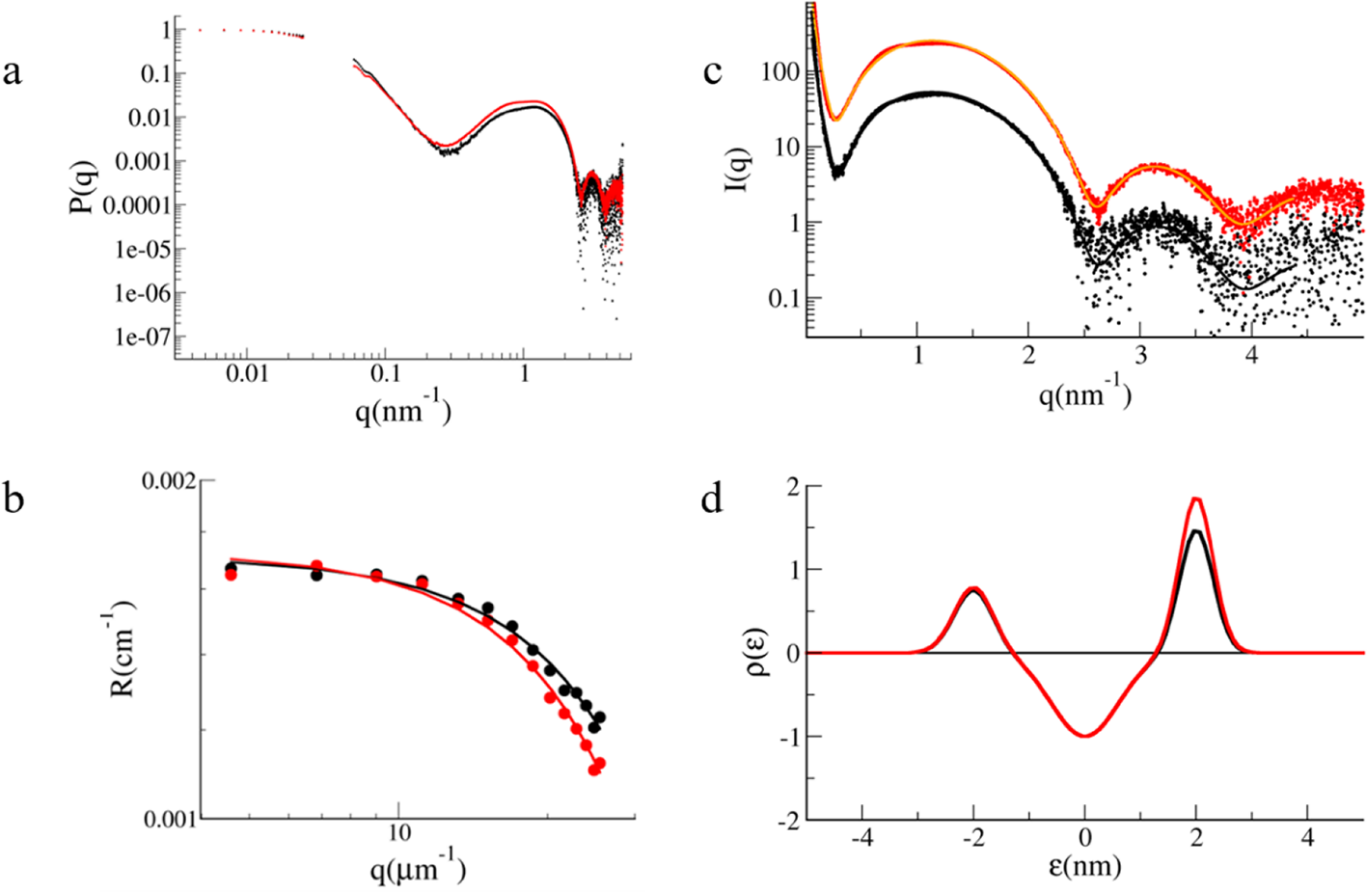
(a) Form factor *P*(*q*) of Lip0 (black) and LipCas (red) obtained by combining data coming from multiangle light and X-ray scattering. (b) LS characterization of liposomes. Rayleigh ratio for Lip0 (black) and LipCas (red); continuous lines represent data fits according to a hollow sphere model. SAXS characterization of liposomes: (c) SAXS data and fitting curves represented by the lines for Lip0 (black) and LipCas (red). (d) Electron density profile obtained as a sum of three Gaussians, corresponding to the best data fits for Lip0 (black) and LipCas (red).

Lip0 and LipCas have been characterized by multiangle light scattering (MALS) and small-angle X-ray scattering (SAXS) to highlight structural differences at different length scales, from vesicle dimension to bilayer properties (Figure 2a). Figure 2a reports the form factor data obtained from light and X-ray scattering, sewed together after suitably scaling the SAXS data in order to obtain the whole liposome form factor.

The analysis of MALS data (Figure 2b) in terms of a hollow sphere with 4 nm thickness gives a radius of 41.5 and 46.5 nm for Lip0 and LipCas, respectively, in reasonable agreement with the DLS and AFM results.^44^

SAXS experiments were performed on Lip0 and unpurified LipCas. Data are reported in Figure 2c and have been analyzed as described in the methods section. A minimal model was used, with only 3 Gaussian curves to describe the shell electronic density, to avoid any bias in the output. The analysis captures well the essential features of the data, such as maxima and nodes, both in position and strength. The resulting electronic densities are reported in the bottom panel of Figure 2d. The asymmetry of the ρ(ε) profile, obtained for both liposomes, is due to the curvature of the nanoparticle, leading to a different arrangement of the polar head in the internal and external layers. The analysis gives the bilayer thickness (3.9 nm for both samples) and the radius of the particles (46 and 52 nm for Lip0 and LipCas, respectively). Figure 2d shows that the two liposomes are mainly different on the external surface, in agreement with the fact that loaded liposomes are more stable in all AFM experiments.

### α_s1_-Casein Loading Efficacy

#### α_S1_-Casein and Liposome Colocalization

The presence of α_S1_-Casein in the liposomes was demonstrated by confocal microscopy. Proteo-liposomes were prepared by loading Alexa Fluor 647-labeled α_S1_-Casein, staining with Di-8-ANEPPS and were observed after the final step of purification by size exclusion chromatography.

**Figure 3:**
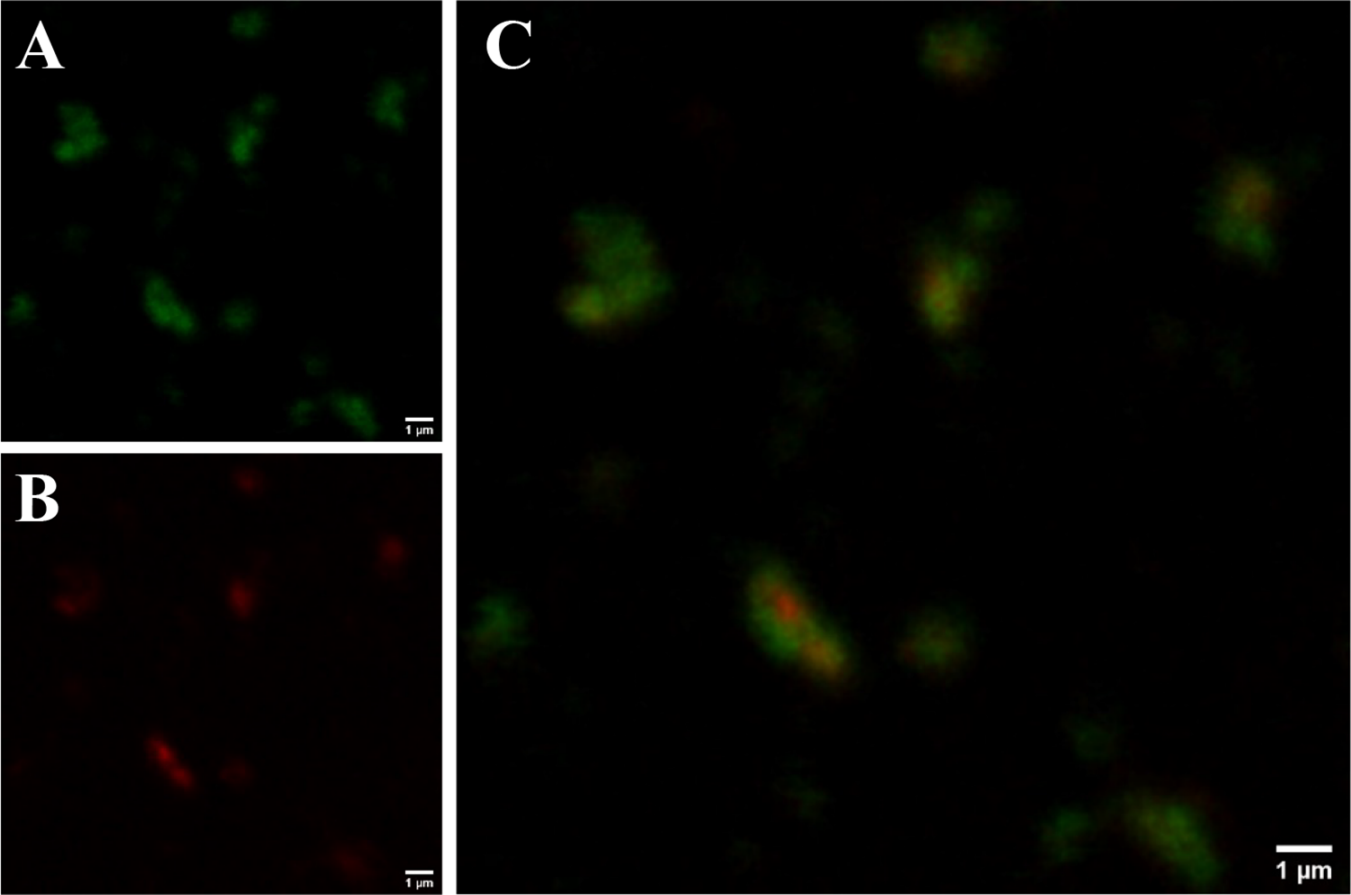
Representative confocal images of α_S1_-Casein loaded proteo-liposomes. (A) Di-8-ANEPPS fluorescence emission channel, acquired in the range 518–650 nm, with λ_exc_= 488 nm. (B) Alexa Fluor 647 fluorescence emission channel, acquired in the range 653–750 nm, with λ_exc_= 633 nm. (C) Overlap of Di-8-ANEPPS and Alexa Fluor 647 fluorescence emission channels. The scale bar is 1 μm.

Figure 3 shows the colocalization of α_S1_-Casein and liposomes. The low resolution does not allow one to distinguish vesicles morphology but only to assess the colocalization of the protein (red signal) with the lipids (green signal). Some vesicle aggregation phenomenon is noticed, likely prompted by the interaction of the proteoliposomes with the sample holder surface.

#### α_S1_-Casein Quantification in LipCas

α_S1_-Casein loaded in liposomes was quantified by using α_S1_-Casein labeled with Alexa Fluor 647 in some analytic preparations. Quantification of α_S1_-Casein was achieved by measuring the emission of purified LipCas-Alexa at λ_em_ = 650 nm by using the standard calibration curve of Alexa Fluor 647 and taking into account the protein degree of labeling previously determined (Figure S1 in the Supporting Information). Conclusively, in a LipCas sample of 0.2 mg/mL lipid concentration, the concentration of the loaded α_S1_-Casein was estimated at 3.8 nM, obtaining a drug loading percentage, DL % = 0.045%, and a loading efficacy percentage, LE % = 0.45%

### *In Vivo* Tests in **C. elegans**

#### Effect of α_s1_-Casein on Aβ Aggregates in **C. elegans**

**Figure 4:**
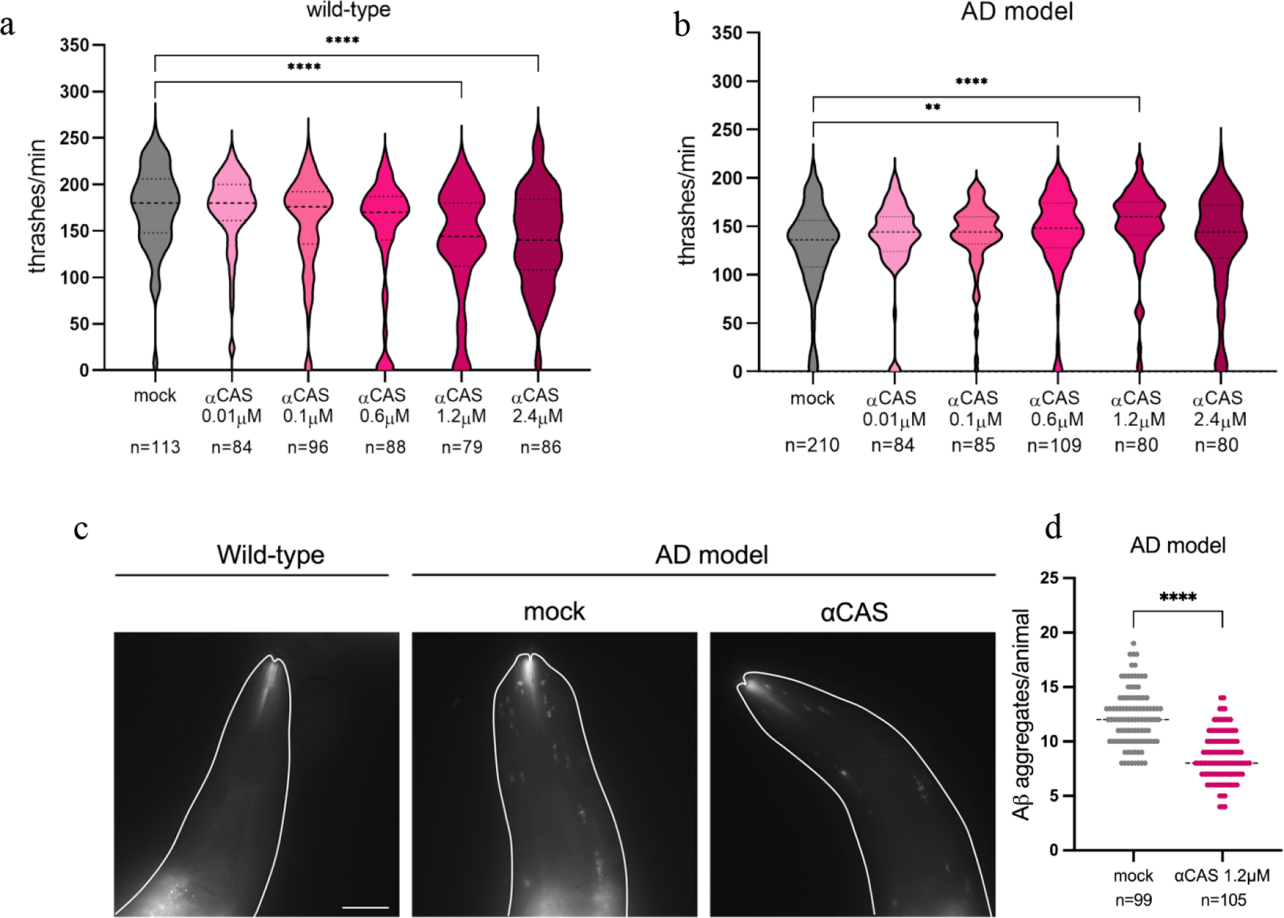
Quantification of locomotion after treatment with different concentrations of α_s1_-Casein. Wild-type animals are on the left (a) and AD animals are on the right (b). Violin plots show the distribution of thrashes performed by the animals in a minute. Bold dashed lines in the center correspond to the median, while upper and lower dashed lines correspond to the quartiles. (c) Representative pictures of wild-type and AD animals heads after staining with X-34, that specifically marks Aβ aggregates. AD animals were pretreated with α_s1_-Casein or mock. Scale bar is 25 μm. (d) Quantification of Aβ aggregates after treatment with α_s1_-Casein (1.2 μM) or mock. Each dot corresponds to the number of visible Aβ aggregates in the head muscles of each animal after X-34 staining. The number of animals scored is reported at the bottom (*n*). One-way ANOVA test (a, b) and Mann–Whitney *t* test (d) were used to determine values significantly different from mock: **p < 0.005; ****p < 0.0001.

To investigate the hindering effect of α_s1_-Casein on amyloid formation in vivo and to test for possible toxic effects, we took advantage of **C. elegans** wild-type and transgenic animals expressing human Aβ_3–42_ peptide specifically in muscles. Aβ aggregates accumulate in muscles and cause an age-dependent defect in locomotion.^31−33^ Wild-type and AD animals have been treated with increasing concentrations of α_s1_-Casein and the thrashing locomotion behavior was scored 3 days after the suspension of the treatment. Interestingly, we observed that only the highest concentrations of α_s1_-Casein (1.2 and 2.4 μM) affect wild-type animal movement inducing a significant decrease in the thrashing behavior compared to untreated animals (Figure 4a). On the other hand, AD animals, which are impaired in the same behavior, showed an improvement in animal motility when treated with the intermediate concentrations of α_s1_-Casein (0.6 and 1.2 μM), while either lower (0.01 and 0.1 μM) or higher (2.4 μM) had no effect (Figure 4b). To better understand the α_s1_-Casein mechanism of action in vivo, we evaluated its effect on Aβ aggregates. To visualize amyloid aggregates in living animals, we used the amyloid-specific X-34 dye, which specifically recognizes amyloid aggregates but not oligomers.^45,46^ X-34 staining highlighted the presence of a large number of Aβ aggregates in the muscles of AD animals, while no aggregates were present in wild-type animals, as expected (Figure 4c). Interestingly, pretreatment of AD animals with α_s1_-Casein at intermediate concentration (1.2 μM) strongly reduced the number of visible Aβ aggregates (Figure 4c,d). Taken together, our results suggest that an excess of α_s1_-Casein is detrimental in wild-type animals, while it can rescue animal motility impairment in pathological conditions at intermediate concentrations by preventing accumulation of Aβ aggregates.

#### Effect of Lip0 and LipCas on **C. elegans** Locomotion

**Figure 5:**
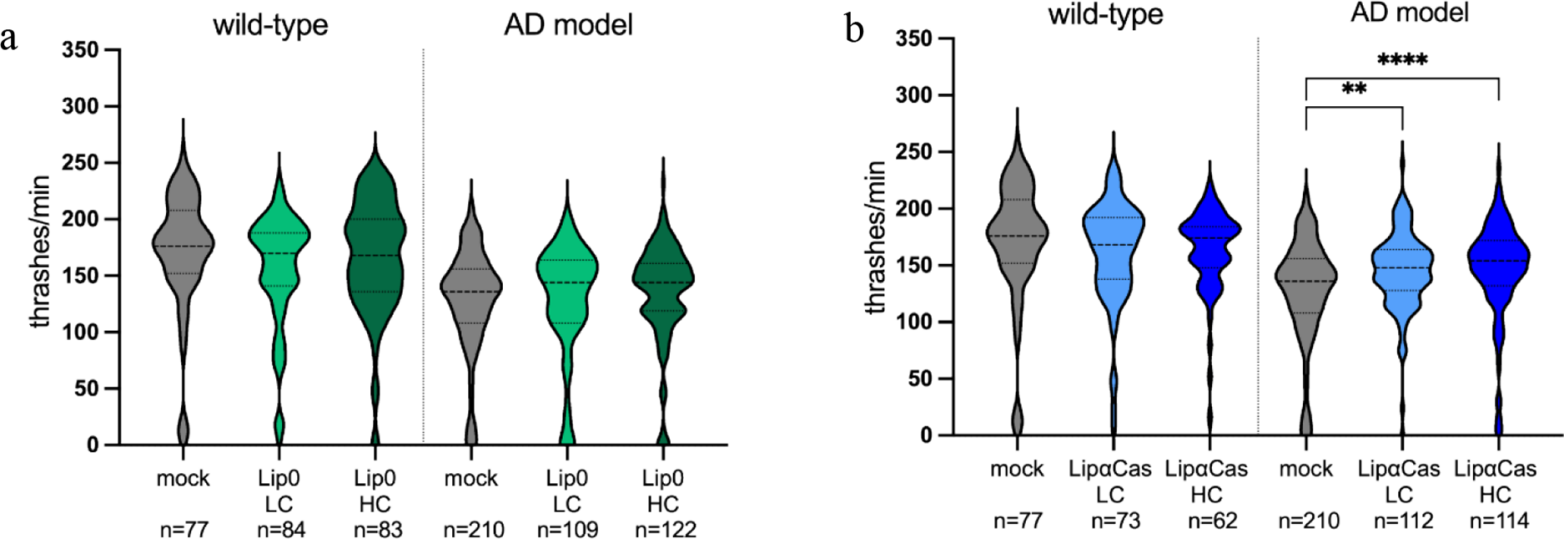
Quantification of locomotion behavior after treatment with Lip0 (a) in wild-type (left) and AD animals (right). The concentrations used (based on lipid content) are LC 21 μg/mL and HC 42 μg/mL. Quantification of locomotion after treatment with LipCas (b) in wild-type (left) and AD animals (right). The concentrations of α_s1_-Casein used are 0.0004 μM for LC and 0.0008 μM for HC. Violin plots show the distribution of thrashes performed by the animals in a minute. Bold dashed lines correspond to the median, while upper and lower dashed lines correspond to the quartiles. The number of tested animals is reported on the bottom for each experiment (*n*). A one-way ANOVA test was used to determine values significantly different from mock: **p < 0.006; ****p < 0.0001.

Since we observed that high concentrations of α_s1_-Casein are detrimental in wild-type animals, we used LUV encapsulation to improve α_s1_-Casein administration and decrease its dose in vivo. We first tested the Lip0 effect on wild-type and AD animals by using two different concentrations of Lip0: a lower concentration (LC, lipid content of 21 μg/mL) and a higher concentration (HC, 42 μg/mL). We did not observe any gross effect on animal fitness and no effect of Lip0 on animal motility (Figure 5a), thus suggesting good biocompatibility of LUV at both concentrations. Therefore, we decided to treat both wild-type and AD animals with LipCas at the same lipid concentrations as above, which correspond to 0.0004 μM (LipCas LC) and 0.0008 μM of α_s1_-Casein content (LipCas HC), respectively, as estimated by the calibration curve (Figure S1). LipCas treatment did not affect the locomotion of wild-type animals, while both concentrations significantly improved AD animal movement (Figure 5b). Thus, LipCas containing 0.0004 μM α_s1_-Casein rescues the locomotion defect (Figure 5b), while free α_s1_-Casein 25 times more concentrated does not (Figure 4b, 0.01 μM). Taken together, our results suggest that LUV encapsulation strongly and efficiently improves the α_s1_-Casein effect in vivo, preventing side effects and significantly reducing the amount of α_s1_-Casein needed to exert its function.

Taken together, in vivo results show the importance of α_s1_-Casein delivery through lipid nanoparticles. The approach of protein encapsulation and purification optimizes the transport of a very tiny amount of α_s1_-Casein, which becomes free to act once delivered. Moreover, thanks to the matrix embedding, self-aggregation as well as unspecific interactions of the protein with the multifaceted cellular environment is minimized.

## Conclusions

Proteo-LUV, incorporating and delivering α_s1_-Casein, has been successfully produced and characterized. Confocal microscopy colocalization of α_s1_-Casein with the lipid bilayer demonstrates successful encapsulation and a drug loading of 0.045% obtained. α_s1_-Casein at very low doses, below nM, administered through unilamellar proteo-liposomes to an AD model of **C. elegans** shows specific effectiveness in partially rescuing animal motility after treatment. It has been initially demonstrated that free α_s1_-Casein at micromolar concentration causes in vivo an impairment of wild-type animal locomotion but rescues the defects observed in AD animals preventing amyloid aggregation in vivo. α_s1_-Casein effects were highly improved when administered as proteo-LUV, with a rescue of the defects in locomotion in AD animals using subnanomolar concentrations and with no toxicity on wild-type animals. Thus, unilamellar vesicle-mediated delivery improves the rescuing capacity of α_s1_-Casein in vivo, allowing the use of more than 10-fold lower concentrations and eliminating any side effect.

As a consequence, the LipCas delivery system represents a proof of concept of the possibility to use partially disordered protein as a therapeutic strategy in AD by liposome encapsulation. The delivery system we propose can thus represent a starting point for the design and development of further stealth liposomes by simple surface PEG-ylation, leading to a product that could be effective, stable in the bloodstream, and suitable for pharmaceutical purposes.^47^

## Methods

### Liposome Preparation

#### Materials

Lyophilized α_s1_-Casein (23.6 kDa) with a purity of 70% was purchased from Sigma-Aldrich Inc. (St. Louis, MO, USA) (cat. C6780) and used without any further purification.^48^ A solution of the protein was prepared in 50 mM phosphate buffer with 20 mM NaCl pH 7.4. The protein concentration was determined by absorption at 280 nm using an extinction coefficient of 0.81 mg^–1^ mL cm^–1^.^49^ The critical micellar concentration in 50 mM buffer phosphate with 20 mM NaCl pH 7.4 was evaluated by using the extrinsic probe ANS fluorescence, obtaining CMC = 0.215 ± 0.005 mg/mL (9.1 μM).

The monounsaturated phospholipids (16:0, 18:1) OPPC (catalog number P4142) and POPS (catalog number 51581) and sheep’s wool cholesterol (catalog number C8667) were purchased from Sigma-Aldrich Inc. and used without any further purification. Sepharose CL-2B was obtained from Sigma-Aldrich Inc. (cat. CL2B300).

#### LUV Preparation

Liposomes were prepared from films obtained by thin layer evaporation of chloroform PC:PS:Chol solutions in a ratio of 85:10:5%w/w. The films were hydrated with a 50 mM phosphate buffer solution with 20 mM NaCl (pH 7.4) or a 0.2 mg/mL α_s1_-Casein phosphate buffer solution to prepare LUV (Lip0) or proteo-LUV (LipCas), respectively. In both preparations, after 1 h of equilibration, the solution underwent at least 5 freeze–thawing cycles in order to achieve full lipid dissolution and also protein incorporation in lipid aggregates. The large unilamellar vesicles were obtained from the multilamellar liposome solution by extrusion, using a polycarbonate membrane filter with a nominal pore size of 50 nm (Avestin, Manheim, Germany), after 31 extrusion steps. The lipid concentration was determined by considering the lipid mass of the initial film and measuring the static light scattering of the extruded solutions as described elsewhere.^50^ In all preparations, the protein concentration was 0.2 mg/mL.

#### α_s1_-Casein Proteo-LUV Purification

High-performance size exclusion chromatography (HPSEC) was performed in order to separate the α_s1_-Casein proteo-LUV from the unentrapped α_s1_-Casein. The purification was carried out using a column (XK 16/20 GE Healthcare Life Science; i.d. 16 mm, length 20 cm) manually packed with Sepharose CL-2B (bed height 16 cm), connected to an ÄKTA PURE system. After column equilibration with 50 mM phosphate buffer solution with 20 mM NaCl (pH 7.4), an isocratic separation has been achieved using the same mobile phase. 500 μL of the proteo-LUV solution has been injected applying a flow rate of 1 mL/min, with a pressure of ca. 2.3 bar at room temperature. The UV detector wavelength was set at 254 nm and fractions of 500 μL were collected.

### α_s1_-Casein Loading Efficacy

#### α_s1_-Casein Labeling Reaction Protocol

αs1-Casein dye conjugate, containing the Alexa 647 Fluor dye (Alexa Fluor 647 Protein Labeling Kit; Invitrogen Corporation, Carlsbad, CA, USA), was prepared according to manufacturers’ instructions. 10.2 mg of αs1-Casein was dissolved in 1 mL of sodium bicarbonate buffer 0.1 M (pH 8.35). A 200 μL portion of protein was added to an aliquot of 100 μg of Alexa 647 Fluor dye. The reaction was stirred for 1h at room temperature. The conjugates were purified from a free dye by size-exclusion chromatography using PD-10 desalting columns packed with Sephadex G-25 resin (GE Healthcare Life Sciences, Buckinghamshire, UK) under gravity. The degree of labeling (DOL) of αs1-Casein dye conjugates was determined spectrophotometrically according to the supplier’s instructions.

#### Method for α_S1_-Casein Quantification in LipCas

The emission spectrum of Alexa Fluor 647 was measured, and a calibration curve for the fluorophore was obtained, as reported in the inset of Figure S1. Afterward, the emission at λ_em_ = 650 nm of Alexa Fluor 647 conjugate α_s1_-Casein proteoliposomes was measured and the concentration of α_S1_-Casein loaded was determined by using the Alexa calibration curve and the degree of labeling previously obtained. The drug loading percentage, defined as , and the loading efficacy percentage, defined as , were obtained. These values were calculated considering that a lipid film of 3 mg was hydrated with 0.5 mL of α_s1_-Casein solution 0.2 mg/mL concentrated, to obtain after the preparation protocol 5 mL of a LipCas solution, 0.2 mg/mL and 0.09 × 10^–3^ mg/mL concentrated in lipid and α_s1_-Casein, respectively.^51^

### Colocalization Experiments

Liposomes extruded with the α_s1_-Casein dye conjugate were stained with the dye Di-8-ANEPPS (ThermoFisher Scientific), whose fluorescence is activated in apolar environments. The staining was performed by incubating liposomes with a 500 nM dye solution, previously filtered through 20 nm filters (Whatmann Anotop), at room temperature for 1 h.^52^ HPSEC has been performed in order to separate the dye-conjugated α_s1_-Casein proteo-LUV from the unentrapped dye conjugated α_s1_-Casein and the free Di-8-ANEPPS. The purification was carried out using a column (XK 16/20 GE Healthcare Life Science; i.d. 16 mm, length 20 cm) manually packed with Sepharose CL-2B (bed height 16 cm), connected to an ÄKTATM PURE system. After column equilibration with 50 mM phosphate buffer solution with 20 mM NaCl (pH 7.4), an isocratic separation has been achieved using the same mobile phase. 500 μL of the proteo-LUV solution has been injected applying a flow rate of 1 mL/min, with a pressure of ca. 2.3 bar at room temperature. The UV detector wavelength was set at 254 nm, and fractions of 500 μL were collected.

α_S1_-Casein loaded proteo-LUV have been imaged using a Leica TSC SP5 confocal laser scanning microscope with a 63× objective, and NA = 1.4. 1024 × 1024 pixel images have been acquired with a sequential acquisition of two channels: Alexa Fluor 647 emission was acquired in the range 653–750 nm, with λ_exc_ = 633 nm; Di-8-ANEPPS emission was acquired in the range 518–650 nm, with λ_exc_ = 488 nm.

### Laurdan Assay

Samples were prepared by mixing Lip0 or LipCas with Laurdan reagent (previously dissolved in DMSO) in the appropriate ratio to obtain a final dispersion containing Laurdan reagent (5 μM) and vesicles (1 mM lipid concentration). After overnight equilibration at 4 °C, the Laurdan emission spectrum was recorded, and the intensity at λ = 435 and 500 nm was used to calculate the Generalized Polarization (GP) as follows:
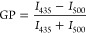


Each experiment was performed in triplicate.

### AFM

Lip0 and LipCas were diluted in 50 mM acetate buffer with 150 mM NaCl (pH 5) to final concentration of 16 μg/mL. 50 μL of the sample was deposited on freshly cleaved mica for 30 min at room temperature and gently rinsed with the same buffer. Quantitative Imaging AFM measurements were performed in this buffer by using a Nanowizard III atomic force microscope (JPK Instruments AG, Germany) equipped with a 100 × 100 × 15 μm^3^ scanner and AC40 (Bruker) silicon cantilever (nominal spring constant 0.1 N/m, nominal tip radius 8 nm), thermally calibrated by using the tool in JPK software.^53^ 2 × 2 μm^2^ images were acquired in a z-closed loop at 256 × 256 pixel resolution (force set point 80 pN, *z* length 50 nm, pixel time 5 ms).

### Light Scattering

Dynamic light scattering (DLS) was used to verify the hydrodynamic size distribution of the LUV and, therefore, the quality of the extrusion. Scattered light intensity and time autocorrelation function were measured using a Brookhaven BI-9000 correlator and a 100 mW solid-state laser at λ = 532 nm. Measurements were performed at *q* = 22.3 μm^–1^. The field autocorrelation functions obtained by DLS were analyzed using a smoothing-constrained regularization method.^54^ The stability of LipCas was studied by static and dynamic light scattering. The purified LipCas resulting was indeed stable for at least 2 weeks, when stored at 8 °C (data not shown).

Multiangle light scattering (MALS) was measured to obtain the form factor of the vesicles, both Lip0 and LipCas, in the range 5 < *q* < 25 μm^–1^. The data were analyzed by using a spherical shell model and by obtaining the mean radius of gyration. The static light scattering data were corrected for the solvent background and normalized by using the scattering intensity of the toluene as reference (*R*_tol_ = 28 × 10^–6^ cm^–1^).

### SAXS

LUV’s small-angle X-ray scattering (SAXS) spectra were recorded at the BL11-NCD beamline of the ALBA Synchrotron Light Facility (Barcelona, Spain). The scattered radiation was recorded by using a two-dimensional CCD detector. The sample–detector distance of 2.175 m covered the momentum transfer interval 0.1 nm^–1^ < *q* < 5 nm^–1^ (*q* = 4π sin (θ)/λ, where 2θ is the scattering angle and λ = 0.124 nm is the X-ray wavelength), the optical path of the X-ray through the sample was about 3 mm. Data were collected from the samples Lip0 and on unpurified LipCas at 20 °C for 10 s. The purification of proteo-LUV results in a strong concentration depletion, which brings about noisy data even after the possible reconcentration steps. The presence of residual protein in solution, however, below the micellar concentration, should not affect the SAXS signal, which in the investigated *q*-range is dominated by the bilayer structure. The CCD camera images taken from the random orientation of vesicles were integrated radially, obtaining a one-dimensional profile of the X-ray scattering intensity *I*(*q*) versus the scattering vector *q*. The buffer scattering intensity was subtracted as a background.

The analysis of the SAXS data was performed by considering as a fitting expression a simplified relation for the intensity, valid for diluted solutions in a suitable range (*q* > 0.1 nm^–1^):
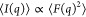
where *F*(*q*) is the Fourier transform of the electronic density ρ(*r*) of the vesicle bilayer, the proportionality accounting for the number of scattering particles as well as the instrumental scaling.

By modeling the electronic density with a combination of three Gaussians and using the Fourier transform of this model function to fit the data, it is possible to extract information about the structure of the bilayer.^55−58^ The three Gaussians account for the electronic density distribution of the two internal and external polar lipid heads and the third one of the central hydrophobic matrix, giving relative contrast with respect to the bulk.

The data were analyzed by considering the analytical expression obtainable by Fourier transformation by assuming the signal coming from perfectly spherical and radially symmetric vesicles of radius *R*:
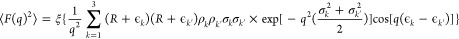
where ρ_*k*_, ϵ_*k*_, and σ_*k*_ are the intensity, the position, and the width, respectively, of the different Gaussian components (3 internal, 1 hydrophobic, 2 externals). The Gaussian representing the hydrophobic interior was taken ϵ_1_ = 0 and ρ_1_ = 1, as described elsewhere.^58^ The bilayer thickness is evaluated as *d* = ϵ_3_ – ϵ_2_, i.e., the distance between the internal and external polar head peaks.

MALS and SAXS data in Figure 2a are sewed according to the expression:
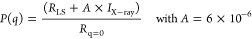


### **C. elegans** Experiments

Nematode growth and maintenance were performed following standard procedures^59^ at 20 °C, on nematode growth medium (NGM) agar plates seeded with *Escherichia coli* strain OP50. Strains used in this work were provided by the *Caenorhabditis* Genetics Center (CGC): N2 (Bristol type) as wild-type animals and CL2120 *dvIs14 [(pCL12) unc-54::beta 3–42 + (pCL26) mtl-2::GFP]* as transgenic animals expressing human Aβ_3–42_ in muscles. Animal treatments with α_s1_-Casein, Lip0, LipCas, and buffer as control were performed *in liquido* in 96-multiwell plates from Falcon (cat. 353072) starting from synchronized eggs, obtained by bleaching, until the young adult stage.^60^ About ∼20 eggs per well in triplicate for each experimental condition have been treated in M9 buffer (3 g KH_2_PO_4_; 6 g Na_2_HPO_4_; 5 g NaCl; 1 mL MgSO_4_ 1M; ddH_2_O to 1 L) with 2× antibiotic/antimycotic solution from Sigma-Aldrich Inc. (cat. A5955), 5 ng/mL cholesterol from Sigma-Aldrich Inc. (cat. C8667), and OP50.^61^ After treatment, animals were transferred to fresh NGM plates seeded with OP50, each day for 3 days. α_s1_-Casein (0.2 mg/mL) was diluted to a final protein concentration of 0.01, 0.1, 0.6, 1.2, and 2.4 μM. Lip0 was used at the following final concentrations of lipid content: LC 21 μg/mL; HC 42 μg/mL. LipCas was used at the following final concentrations of α_s1_-Casein: LC 0.4 nM; HC 0.8 nM and similar lipid content of Lip0. For the thrashing assay, animals were transferred in 7 μL of M9 buffer in a sterile clock glass, left for 5 min in the buffer, and then video-recorded for 30 s. The measurement of thrashes was done by counting every change of direction with respect to the longitudinal axis of the body and multiplying by two. For Aβ aggregate staining, animals have been pretreated with mock or α_s1_-Casein (1.2 μM) as described above. After 72h, the treatment was interrupted and animals transferred to fresh NGM plates seeded with OP50. After 48h, animals were stained with 200 μM 1,4-bis(3-carboxy-hydroxy-phenylethenyl)-benzene (X-34), Sigma-Aldrich Inc. (cat. SML1954), in 10 mM Tris-HCl buffer pH 8.0 for 2h at RT followed by 16 h destaining.^62^ For microscopy analysis, animals were immobilized with 0.01% tetramisole hydrochloride, Sigma-Aldrich Inc. (cat. T1512), on 4% agar pads. The number of visible Aβ aggregates in the head muscles of the animal was quantified using a Zeiss Axioskop microscope using a 40× objective and DAPI filter. Epifluorescence images were collected with a Leica TCS SP8 AOBS microscope. One-way ANOVA (Kruskal–Wallis multiple comparison test) and Mann–Whitney *t* test statistical analysis were performed with GraphPad Prism and *p* < 0.05 used as the threshold for statistically significant differences between groups.

## Supporting Information Available

Fluorescence emission spectrum of LipCas-Alexa (PDF)
Thrashing behavior of wild-type animals treated with mock (AVI)
Thrashing behavior of AD animals treated with mock (AVI)
Thrashing behavior of AD animals treated with LipCas HC(AVI)

## Supplementary Material

cn3c00239_si_001.pdf

cn3c00239_si_002.avi

cn3c00239_si_003.avi

cn3c00239_si_004.avi

## ABBREVIATIONS

OPPC: (1-oleoyl-2-palmitoyl-*sn*-glycero-3-phosphatidylcholine)
POPS: (1-palmitoyl-2-oleoyl-*sn*-glycero-3-phospho-l-serine)
APL-1: (amyloid Precursor-Like-1)
*C. elegans*: (*Caenorhabditis elegans*)
