# Supplementary material for: αS1-Casein-Loaded Proteo-liposomes as Potential Inhibitors in Amyloid Fibrillogenesis: In Vivo Effects on a C. elegans Model of Alzheimer’s Disease
Source: ACS Chem Neurosci. 2023 Oct 17;14(21):3894–904. doi: 10.1021/acschemneuro.3c00239 (PMC10623563; doi:10.1021/acschemneuro.3c00239)
Supplement: Supplementary file 1 — cn3c00239_si_001.pdf [file cn3c00239_si_001.pdf]

Supporting Information

α_{S1}-Casein-loaded proteo-liposomes as potential inhibitors in Amyloid fibrillogenesis: in vivo effects on a *C. elegans* model of Alzheimer's disease

Angela Paterna[1#], Pamela Santonicola[2,3#], Giulia Di Prima[4], Estella Rao[1], Samuele Raccosta[1], Giuseppina Zampi[2], Claudio Russo[3,5], Oscar Moran[6], Mauro Manno[1], Elia Di Schiavi[2], Fabio Librizzi[1] and Rita Carrotta[1*]

[1]Institute of Biophysics, National Research Council, Division of Palermo, Via Ugo La Malfa 153, 90146, Palermo, Italy

[2]Institute of Biosciences and Bioresources, Division of Napoli, Via Pietro Castellino 111, 80131, Napoli, Italy

[3]Department of Medicine and Health Sciences, University of Molise, 86100 Campobasso, Italy

[4]Department of Biological, Chemical and Pharmaceutical Sciences and Technologies, University of Palermo, 90123 Palermo, Italy

[5]Consorzio Interuniversitario in Ingegneria e Medicina (COIIM), Via F. De Sanctis, 86100 Campobasso, Italy

[6]Institute of Biophysics, National Research Council, Division of Genova, Via De Marini 6, 16149, Genova, Italy

# These authors have equally contributed

*Correspondence: Dr. Rita Carrotta, +39-0916809313, rita.carrotta@cnr.it

Supplementary Figure

The emission spectrum of purified LipCas-Alexa at λ_{em}= 650 nm is reported together with the standard calibration curve of Alexa Fluor 647 in Fig.S1.

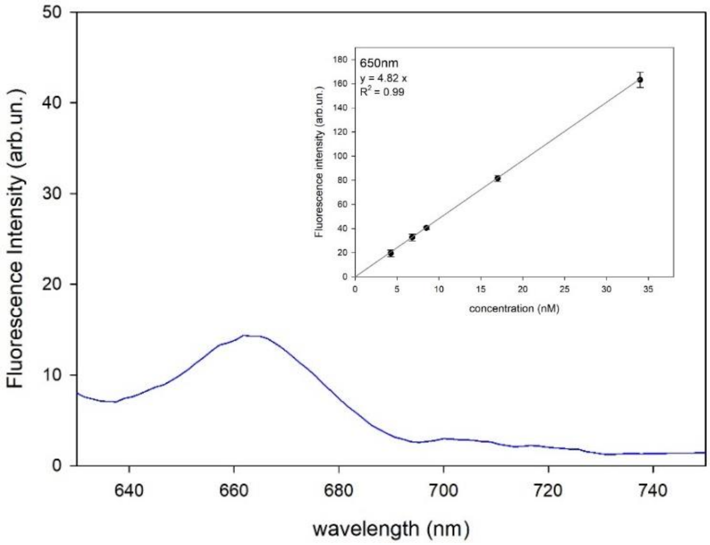

Figure S1: Fluorescence emission spectrum of LipCas at 0.2 mg/ml. Spectrum has been acquired at room temperature in the range 630-750 nm, with λ_{exc} = 600 nm. The calibration curve of Alexa Fluor 647 is shown in the inset, plotting the intensity of the fluorescence at 650 nm versus the concentration (nM) of Alexa Fluor 647.

Supplementary Videos

Three representative videos (avi) showing the thrashing behavior of wild-type animals treated with mock (video 1), AD animals treated with mock (video 2) and AD animals treated with LipCas HC (video 3) are included as supplementary information.

Here the captions to the videos:

Sup. video 1: Representative video showing the thrashing behavior of 5 wild-type *C. elegans* animals treated with mock. The average number of thrashes per animal in this video of 30 seconds is 87.6.

Sup. video 2: Representative video showing the thrashing behavior of 5 AD *C. elegans* animals treated with mock. The average number of thrashes per animal in this video of 30 seconds is 56.4.

Sup. video 3: Representative video showing the thrashing behavior of 5 AD *C. elegans* animals treated with LipCas HC. The average number of thrashes per animal in this video of 30 seconds is 72.8.
